# Egg Laying of Cabbage White Butterfly (*Pieris brassicae*) on *Arabidopsis thaliana* Affects Subsequent Performance of the Larvae

**DOI:** 10.1371/journal.pone.0059661

**Published:** 2013-03-19

**Authors:** Sven Geiselhardt, Kinuyo Yoneya, Beatrice Blenn, Navina Drechsler, Jonathan Gershenzon, Reinhard Kunze, Monika Hilker

**Affiliations:** 1 Institute of Biology – Applied Zoology/Animal Ecology, Freie Universität Berlin, Berlin, Germany; 2 Institute of Biology – Applied Genetics, Freie Universität Berlin, Berlin, Germany; 3 Department of Biochemistry, Max Planck Institute for Chemical Ecology, Jena, Germany; Centro de Investigación y de Estudios Avanzados, Mexico

## Abstract

Plant resistance to the feeding by herbivorous insects has recently been found to be positively or negatively influenced by prior egg deposition. Here we show how crucial it is to conduct experiments on plant responses to herbivory under conditions that simulate natural insect behaviour. We used a well-studied plant – herbivore system, *Arabidopsis thaliana* and the cabbage white butterfly *Pieris brassicae*, testing the effects of naturally laid eggs (rather than egg extracts) and allowing larvae to feed gregariously as they do naturally (rather than placing single larvae on plants). Under natural conditions, newly hatched larvae start feeding on their egg shells before they consume leaf tissue, but access to egg shells had no effect on subsequent larval performance in our experiments. However, young larvae feeding gregariously on leaves previously laden with eggs caused less feeding damage, gained less weight during the first 2 days, and suffered twice as high a mortality until pupation compared to larvae feeding on plants that had never had eggs. The concentration of the major anti-herbivore defences of *A. thaliana*, the glucosinolates, was not significantly increased by oviposition, but the amount of the most abundant member of this class, 4-methylsulfinylbutyl glucosinolate was 1.8-fold lower in larval-damaged leaves with prior egg deposition compared to damaged leaves that had never had eggs. There were also few significant changes in the transcript levels of glucosinolate metabolic genes, except that egg deposition suppressed the feeding-induced up-regulation of *FMO_GS-OX2_*, a gene encoding a flavin monooxygenase involved in the last step of 4-methylsulfinylbutyl glucosinolate biosynthesis. Hence, our study demonstrates that oviposition does increase *A. thaliana* resistance to feeding by subsequently hatching larvae, but this cannot be attributed simply to changes in glucosinolate content.

## Introduction

Plants are well known to use cues to anticipate attack by herbivorous insects and enhance their defensive posture. For example, after perceiving herbivore-induced volatiles emitted from an already infested part of the plant or infested neighbouring plants. Both direct and indirect plant defensive responses are commonly enhanced [1]–[6].

Another potential predictor of future insect attack is when eggs are laid on plants. A wide range of studies has shown that plants are able to react to the presence of insect eggs by (i) direct defences that harm the eggs [7], [8] and (ii) by indirect defences that attract egg parasitoids to egg-induced leaf volatiles [9]–[12] or arrest parasitoids by egg-induced changes of leaf surface chemistry [13], [14].

Plants also appear to respond to insect eggs by producing direct defences active against subsequent feeding stages. For example, herbivorous pine sawfly larvae (*Diprion pini* (L.)) that fed on previously egg-laden twigs of *Pinus sylvestris* L. gained much less weight and suffered significantly higher mortality than sawfly larvae fed on egg-free pine twigs [15]. Furthermore, infestation of tomato leaves (*Solanum lycopersicum* L.) by adults of the bug *Orius laevigatus* Fieber, which insert eggs into tomato leaf tissue, resulted in a jasmonic acid (JA)-mediated wound response that lowered subsequent feeding damage by the western flower thrips *Frankliniella occidentalis* (Pergande); in contrast, infestation of tomato leaves by *O. laevigatus* nymphs (which do not lay eggs) had no such effect [16]. Moreover, egg deposition by the tomato fruitworm moth *Helicoverpa zea* Boddie on tomato leaves caused a burst of jasmonic acid and primed the feeding-induced up-regulation of a gene encoding a proteinase inhibitor (*pin2*) [17].

In contrast, Bruessow et al. [18] showed that treatment of *Arabidopsis thaliana* (L.) Heynh. leaves with extracts from crushed eggs of the butterfly *Pieris brassicae* (L.) had no effect on the weight gain of conspecific larvae feeding on these leaves for 8 days, and larvae of the generalist *Spodoptera littoralis* Boisd. actually gained more weight on treated leaves compared to untreated leaves. However, it is still unknown whether treatment of leaves with egg extracts induces the same effects on the plants response to feeding larvae as natural egg deposition. Moreover, in the study of Bruessow et al. [18] *P. brassicae* was tested as individually feeding larvae, although this species naturally feeds gregariously. In addition, the effect of egg extracts on parameters of insect performance other than larval weight was not studied.

The limitations of this previous work and our finding that natural egg deposition by *P. brassicae* on *A. thaliana* leaves can induce indirect plant defence against the eggs [14] prompted us to test the hypothesis that egg deposition by this insect also affects direct plant defence against the larvae. Hence, we first investigated (i) if natural egg deposition by *P. brassicae* can alter feeding behaviour and reduce the performance of conspecific larvae that were allowed to feed gregariously after hatching. Under natural conditions, freshly hatched larvae first feed on their egg shells before consuming plant tissue, so we also determined (ii) if access to the egg shells affects performance of young larvae and extent of leaf damage caused by them.

As direct defences, we investigated the levels of glucosinolates (GLS) in intact and feeding-damaged *A. thaliana* leaves with and without prior egg deposition. GLS are the best known group of anti-herbivore defences in the family Brassicaceae against a broad range of enemies [19]–[21]. Stored in plants as glycosides, they are activated on plant damage by myrosinases and other proteins to form a variety of potent hydrolysis products. We asked whether egg deposition by *P. brassicae per se* affects (iii) the glucosinolate content of *A. thaliana* and (iv) the transcript levels of genes involved in GLS biosynthesis and activation.

## Results

### Larval performance

Under natural conditions, neonate larvae start feeding on their egg shell before they consume leaf tissue. However, access to the egg shells during the first two days of larval feeding did not affect weight and mortality of young larvae (Table 1 and 2). On the other hand, prior egg deposition on a plant had significant effects on the extent of larval feeding and on larval performance. Freshly hatched larvae consumed less leaf tissue (rANOVA, *F_1,14_*  = 6.00, *P* = 0.03) and gained less weight (rANOVA, *F_1,13_*  = 10.73, *P* = 0.006) during the first two days after hatching when they fed on previously egg-laden leaves compared to egg-free leaves (Table 1 and 2). However, the mortality of the young larvae was similar in both treatment groups (rANOVA, *F_1,14_*  = 0.004, *P*>0.05; Table 1 and 2).

**Table 1:** Effects of prior egg deposition and egg shell consumption (a typical behaviour of neonate larvae) on larval performance (means ± SE) of *Pieris brassicae* on *Arabidopsis thaliana* Col-0 plants^1^ (for statistics, see Table 2).

Parameter		Access to egg shells^2^			No access to egg shells^3^		
		Control	Egg	*N* ^4^	Control	Egg	*N* ^4^
Consumed leaf area after 2 days (cm^2^)		3.27±0.49	2.52±0.33	8	2.82±0.17	2.26±0.27	8
Larval weight (2d-old) (mg)		0.36±0.03	0.32±0.03	8	0.33±0.01	0.29±0.02	7
Mortality (%)	from hatching to 2^nd^ day	16.3±4.9	16.6±3.3	8	20.9±4.2	20.9±3.9	8
	from 4^th^ day to pupation	40.0±13.2	60.0±20.0	4	20.0±10.8	55.0±16.6	4

1 Batches of 40 freshly hatched larvae either fed upon a plant with prior *P. brassicae* egg deposition (Egg) or without any eggs (Control) until they were 4 days old; thereafter, batches of 10 larvae where transferred to fresh, undamaged egg-free plants, where they completed their development until pupation. ^2^ Larvae were allowed to feed upon their egg shells. ^3^ Larvae were prevented from feeding upon their egg shells during the first 2 days after hatching. ^4^ Number of batches of larvae (1 batch per plant; *N* = 8 for freshly hatched larvae; *N*  =  initially 4 for elder larvae).

**Table 2:** ANOVA statistics for effects of prior egg deposition and consumption of egg shells on the larval performance of *Pieris brassicae* on *Arabidopsis thaliana* Col-0 plants (experimental data in Table 1).

Parameter measured	Effect	*F*	*df*	*P*
Consumed leaf area after 2 days	Egg shell	0.93	1,14	0.35
	Egg deposition	6.00	1,14	**0.03**
	Egg shell x egg deposition	0.12	1,14	0.73
Larval weight (2d-old)	Egg shell	0.97	1,13	0.38
	Egg deposition	10.73	1,13	**0.006**
	Egg shell x egg deposition	0.02	1,13	0.88
Mortality from larval hatching to 2^nd^ day	Egg shell	0.79	1,14	0.39
	Egg deposition	0.00	1,14	0.95
	Egg shell x egg deposition	0.00	1,14	0.95
Mortality from 4^th^ larval day to pupation	Egg shell	0.55	1,6	0.48
	Egg deposition	7.41	1,6	**0.03**
	Egg shell x egg deposition	0.55	1,6	0.49

After 4 days of feeding on previously egg-laden plants or egg-free plants, larvae were transferred to egg-free control plants, since their original host plants were almost completely defoliated. This experimental manipulation reflects the natural situation since larvae of *P. brassicae* and other species frequently leave host plants that no longer provide sufficient food and search for a new host. The experience of feeding on previously egg-laden plants for the first 4 days negatively affected later survival of larvae subsequently fed on egg-free plants. Their mortality before pupation was almost twice as high as the mortality of larvae which started their development on egg-free control plants (rANOVA, *F_1,6_*  = 7.4, *P* = 0.03; Table 1 and 2).

### Glucosinolate (GLS) content

Four aliphatic GLS [glucoiberin (3MSOP), glucoraphanin (4MSOB), glucoalyssin (5MSOP), and glucohirsutin (8MSOO)] and two indole GLS [glucobrassicin (I3M) and 4-methoxyglucobrassicin (4MO-I3M)] were detected in the leaves of *A. thaliana* (Table 3). Egg deposition *per se* had no effect on GLS concentration (Table 3). Neither the total GLS content nor concentrations of individual GLS differed between undamaged, egg-free control leaves (‘C’ leaves) and leaves laden with eggs for 5 days (‘E’ leaves). The short 2-day-period of feeding by freshly hatched larvae on egg-free leaves (‘F’ leaves) led to a slight increase of the indolic I3M by about 25% compared to ‘C’ leaves (Fisher's LSD, *P*<0.05), but other GLS remained unaffected (Table 3). However, after 2 days of larval feeding, the total GLS concentration was significantly lower in previously egg-laden leaves (‘E+F’) than in leaves that did not have eggs before feeding (‘F’) (Table 3; Fisher's LSD, *P*<0.001). This effect was mainly due to lower amounts of the short-chained aliphatic 3MSOP and 4MSOB in ‘E+F’ leaves than in ‘F’ leaves (Table 3; Fisher's LSD, *P*<0.05). The concentrations of other GLS in feeding-damaged leaves were not affected by prior egg deposition (Table 3).

**Table 3:** Mean (± SE) glucosinolate content (μmol/g dry weight) of *Arabidopsis thaliana* Col-0 plants subjected to different *Pieris brassicae* feeding and egg-laying treatments.

Glucosinolate^1^	Plant treatment^2^																					
	C						E					E+F										F
Aliphatic																						
3MSOP	1.06	±		0.09^ab^		0.99			±	0.13^ab^			0.81		±	0.09^a^		1.19	±			0.10^b^
4MSOB	6.46	±		0.73^a^		5.70			±	0.93^ab^			4.09		±	0.49^b^		7.29	±			0.63^a^
5MSOP	0.34	±		0.06^a^		0.26			±	0.03^a^			0.25		±	0.03^a^		0.35	±			0.04^a^
8MSOO	0.43	±		0.03^a^		0.39			±	0.04^a^			0.39		±	0.04^a^		0.40	±			0.03^a^
Indolic																						
I3M	1.27	±		0.11^a^		1.36			±	0.12^ab^			1.58	±		0.11^ab^		1.60	±	0.12^b^		
4MOI3M	0.89	±		0.05^a^		0.96			±	0.05^a^			0.99	±		0.07^a^		0.83	±	0.06^a^		
Total	10.46		±		0.89^ab^	9.66		±			1.17^ab^	8.11		±		0.79^a^	11.65		±		0.69^b^	

1 Abbreviation of glucosinolates: 3MSOP: 3-methylsulfinylpropyl, 4MSOB: 4-methylsulfinylbutyl, 5MSOP: 5-methylsulfinylpentyl, 8MSOO: 8-methylsulfinyloctyl, I3M: indol-3-ylmethyl, 4MOI3M: 4-methoxyindol-3-ylmethyl.

2 Treatments: C: leaves of untreated intact plants (*N* = 14); E: leaves of plants on which eggs were laid and left for 5 days (*N* = 14); E+F: leaves of plants on which eggs were laid then larvae hatched and fed for 2 days (*N* = 14); F: leaves of plants infested by larvae for 2 days, no eggs laid on plants (*N* = 14).

Significant differences (*P*<0.05) between treatments (within a row) are indicated by different letters; MANOVA with *post hoc* Fisher's LSD tests.

### Expression of genes related to glucosinolate metabolism

**Figure 1:**
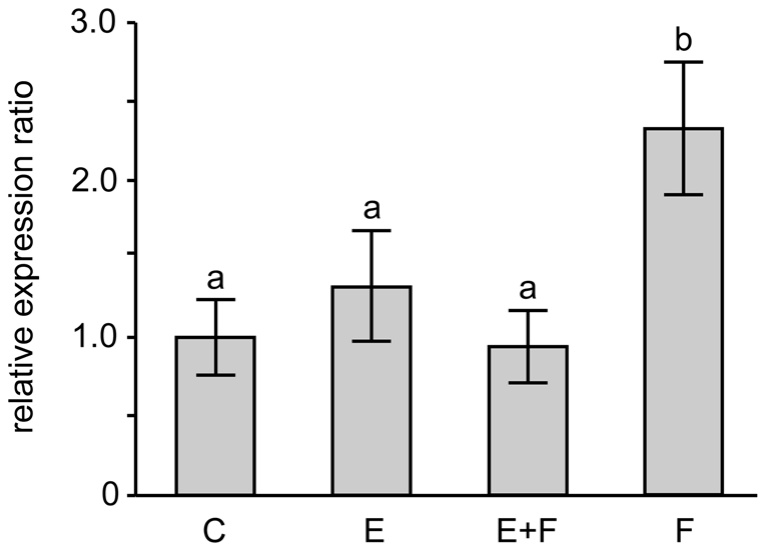
Expression ratios of *FMO_GS-OX2_* in leaves subjected to different oviposition and feeding treatments. Values are means ± standard errors of wild-type *Arabidopsis thaliana* plants (Col-0). C: untreated control leaves (*N* = 8); E: leaves on which eggs were laid and left for 5 days (*N* = 8); E+F: leaves on which eggs were laid and caterpillars hatched and fed for 2 days (*N* = 7); F: leaves that never had eggs but were fed on for 2 days (*N* = 7). Data were normalised to the amplification of ubiquitin, calibrated against the value of the control, and statistically evaluated by analyses of variance (ANOVA). Different letters above the columns indicate significant differences by means of Fisher's LSD test for *post hoc* comparisons (*P*<0.05).

To investigate whether natural egg deposition modulates the plants molecular response to gregariously feeding larvae, we studied a set of 30 genes involved in GLS biosynthesis, regulation of biosynthesis and activation by hydrolysis [19]–[22]. When comparing feeding-damaged leaves with and without eggs, we found significantly different transcript levels for *FMO_GS-OX2_*, a gene encoding an enzyme that catalyses the final step of 4MSOB biosynthesis (Figure 1). Expression of *FMO_GS-OX2_* was 2.3-fold lower in ‘E+F’ leaves than in ‘F’ leaves (Figure 1; Fisher's LSD, *P*<0.01). This reduced expression was consistent with the lower 4MSOB concentration in ‘E+F’ leaves as compared to ‘F’ leaves (Table 3). While larval feeding on egg-free leaves (‘F’ leaves) led to a significant increase in the transcript levels of *FMO_GS-OX2_* (Figure 1; Fisher's LSD, *P*<0.01), prior egg deposition significantly attenuated this feeding-induced increase.

Neither egg deposition nor feeding had any effect on the expression of all the other GLS genes studied except that the expression of the nitrile specifier protein genes was up-regulated 1.5- to over 5-fold by feeding (Table S1). The nitrile specifier proteins direct hydrolysis of glucosinolates to nitriles instead of isothiocyanates [23].

## Discussion

### Effects of egg deposition on insect performance

In laboratory studies of plant defences with intact plants, herbivorous larvae are typically placed on egg-free leaves (e.g. [24], [25]). However, in nature egg laying often precedes feeding by newly hatched larvae, so we investigated the effect of prior egg deposition on the performance of *P. brassicae* larvae feeding on *A. thaliana*. Our results show that the feeding, growth and survival of *P. brassicae* larvae was negatively affected by prior egg deposition. Two-day-old larvae fed on previously egg-laden *A. thaliana* leaves inflicted less feeding damage to the plant and gained less weight compared to larvae reared on egg-free leaves (Table 1). Moreover, the mortality until pupation of larvae that started feeding on leaves that had eggs laid on them was about twice as high as that of larvae starting on egg-free leaves. Similar results were obtained for the pine sawfly *D. pini* on *P. sylvestris* [15]. Sawfly larvae that start their larval development on previously egg-laden pine twigs perform significantly worse than those on egg-free twigs. Hence, larvae may suffer heavily from the effects of prior egg deposition on a plant, even if they leave the egg-laden plant only a few days after larval hatching and switch to an egg-free plant.

This conclusion contrasts with the one by Bruessow et al. [18] who found no effects of leaf treatment with extracts of crushed eggs on the weight of singly feeding *P. brassicae* larvae after an 8-day-feeding period on *A. thaliana* leaves. The authors concluded from these data that egg deposition on *A. thaliana* has no effects on the larval performance of this herbivore specialist, but did not record any other parameters of performance besides weight. Differences in the experimental design of the studies by Bruessow et al. [18] compared to our studies might have led to the different outcomes. The extracts of crushed eggs used by Bruessow et al. [18] might cause plants to respond to larval feeding in a different way than natural egg deposition as we used. Moreover, plant defensive responses to singly feeding larvae as used by Bruessow et al. [18] might differ from the natural situation of gregariously feeding larvae (*N = *40 freshly hatched larvae per leaf as employed in our study).

### Effects of egg deposition on transcription of genes involved in GLS biosynthesis

Egg deposition by *P. brassicae* suppressed feeding-induced transcription of *FMO_GS-OX2_* which encodes a flavin-monooxygenase catalyzing the S-oxygenation of methylthioalkyl- to methylsulfinylalkyl-GLS independent of chain length, i.e. the final step in the biosynthesis of 3MSOP and 4MSOB [26], [27]. Interestingly, the suppressed expression of *FMO_GS-OX2_* in feeding-damaged leaves with prior eggs corresponds with the lower concentrations of 3MSOP and 4MSOB in these leaves. The decrease in both *FMO_GS-OX2_* transcript and short-chained methylsulfinylalkyl-GLS suggests that levels of their immediate methylthioalkyl-precursors, 3-methylthiopropyl GLS (3MTP) and 4-methylthiobutyl GLS (4MTB), usually intermediates present in only low amounts, might be elevated in egg-laden, feeding-damaged (‘E+F’) leaves [27]. However, we were not able to reliably detect 3MTP or 4MTB in any of the samples of this study.

Transcript levels of most of the other genes of GLS biosynthesis and activation measured did not show significant differences between feeding and egg-laying treatments (Table S1, ‘E+F’‘/F’). The increase in the expression of nitrile specifier protein genes observed after *P. brassicae* feeding on *A. thaliana* (Table S1) has precedence in the literature. *P. rapae* larvae feeding on *A. thaliana* increased the expression of these same genes [23] which increased the proportion of nitriles to isothiocyanates formed upon glucosinolate hydrolysis. This is presumably a strategy of *A. thaliana* against adapted herbivores, such as *Pieris* species. Both species, *P. brassicae* and *P. rapae*, are able to avoid the toxicity of glucosinolates by producing their own specifier proteins to divert isothiocyanate formation to nitriles [28], [29]. Plant production of nitriles instead of isothiocyanates, as indicated by the increase in nitrile specifier protein gene transcripts, does not impair *P. rapae* larval performance, but decreases future oviposition rates and increases the attraction of natural enemies [30].

Surprisingly, none of the gene transcripts measured in this study was affected by egg deposition *per se.* In contrast, Little et al. [31] reported that egg deposition by *P. brassicae* on *Arabidopsis* triggered transcript changes of a broad set of genes in leaf tissue below an egg mass. For example, they found that some of the genes that are involved in the biosynthesis of indolic GLS (*CYP79B2*, *CYP83B1*, *SUR1*) were up-regulated 3 days after egg deposition, while we observed no changes in transcript levels of these genes 5 days after oviposition (Table S1). Little et al. [31] analysed transcript levels in leaf tissue right below an egg mass, while we examined tissue that was not situated below the egg mass, but was adjacent to it since this is the tissue that is consumed by young larvae after hatching. Thus, the findings by Little et al. [31] and those presented here indicate that egg-induced changes of transcript levels of these genes may depend on the time elapsed since egg deposition on a leaf and/or on the distance of analysed leaf tissue to an egg clutch.

### Effects of egg deposition on GLS concentration

Glucosinolate concentrations of *A. thaliana* leaves were not substantially affected by *P. brassicae* oviposition. Nevertheless, prior oviposition significantly altered the accumulation of the short-chained aliphatic 3MSOP and 4MSOB in response to larval damage. Feeding-damaged leaves with prior eggs (‘E+F’ leaves) showed a 1.5- and 1.8-fold reduction in their levels of 3MSOP and 4MSOB, respectively, when compared to egg-free, but feeding-damaged (‘F’) leaves (Table 3). While some previous studies showed an induction of GLS in response to *P. brassicae* or *P. rapae* feeding, other studies showed no increase [21]. In the only previous study involving a *Pieris* species feeding on *A. thaliana*, no GLS induction was observed [32]. The lack of any increase in GLS concentration following *P. brassicae* oviposition in our work means that this class of plant defences does not account for the reduced feeding, growth and survival of feeding larvae observed after oviposition in comparison to feeding on egg-free plants. Oviposition may instead have led to other changes that impaired larval performance, such as increases in the levels of proteinase inhibitors induced upon subsequent larval feeding [17].

### Conclusion

Plants are commonly observed to increase their defences after herbivore attack, but this strategy may not always be effective against a specialist feeder, such as *P. brassicae*. As already mentioned, *P. brassicae* has biochemical adaptations to avoid the toxicity of certain glucosinolate hydrolysis products [28], [29]. Since females of *P. brassicae* lay egg clusters with 10–100 eggs, the gregariously feeding larvae could defoliate the original plant within a few days, and will subsequently move to a neighbouring plant [33]. Thus, it may be beneficial to invest in strategies other than chemical defence to alleviate the effects of herbivory by specialist insects. It was recently demonstrated that oviposition of *P. brassicae* leads to accelerated seed production in black mustard *Brassica nigra* L. [34]. Our study showed that larvae which fed on an egg-laden plant for 4 days and then switched to an egg-free plant suffered significantly higher mortality until pupation than larvae starting their life on an egg-free plant. Up to now, it is unknown how egg-laden *A. thaliana* plants enhance the mortality of *P. brassicae* larvae and whether these plants benefit from an egg-mediated altered response to insect feeding. Future investigations should address the mechanism by which prior oviposition on *A. thaliana* affects performance of *P. brassicae* larvae, ideally by employing natural egg deposition and larval feeding behaviour. Mimicking natural conditions provides the best chance of determining whether a plant actually benefits from an ability to perceive insect egg deposition and to modify its responses to subsequent feeding damage (compare e.g. [35]).

## Materials and Methods

### Plants and insects

*Arabidopsis thaliana* (L.) Heynh. ecotype Columbia (Col-0) plants were used for the insect performance experiments and plant chemical analysis. *A. thaliana* seeds (obtained from continuous culture at the Max-Planck-Institute of Chemical Ecology in Jena, Germany) were sown on standard potting soil (Einheitserde Typ T, Kausek GmbH and Co. KG, Mittenwalde, Germany) with addition of vermiculite (Kausek GmbH and Co. KG, Mittenwalde, Germany), stratified for 3 days at 4°C and grown in a climate chamber under short day conditions (22±1°C, 70±5% RH, L10:D14).

*Pieris brassicae* (L.) larvae were reared on Chinese cabbage (*Brassica rapa* L. ssp. *pekinensis* cv. Kantonner Witkrop) in a climate chamber (20±1°C, 70±5% RH, L18:D6). At these climate conditions, the egg stage takes 6 days and the larval phase about 21 days (with 5 instars).

### Treatment of plants: General

Test plants were plants that (i) received *P. brassicae* eggs (‘E’) or (ii) were left without eggs, but exposed to larval feeding damage (‘F’), or (iii) were subjected to both egg deposition and feeding (‘E+F’). Control plants (‘C’) were left untreated. The total numbers of test plants and egg-free control plants used for the ecological studies (insect performance, plant damage) are given in Table 1, and those for determination of glucosinolate concentrations and transcription rates are given in Table 3, Figure 1 and Table S1. All plants used in experiments were in the rosette stage. The experiments were carried out between September 2009 and March 2010.

### Treatment of plants with eggs

In order to obtain egg-laden test plants, 7 to 11 weeks after germination *A. thaliana* plants were placed singly into a cage (80×100×80 cm). Adults of *P. brassicae* (100 individuals of both sexes) were added to the cage for about 24 h so females could oviposit onto the plant. Thereafter, egg-laden plants with about 3 clusters with 30–50 eggs each (‘E’ treatment) were placed into a climate-controlled chamber (20±1°C, 70±5% RH, (L18:D6). Simultaneously, undamaged control (‘C’) plants that had never had eggs were placed into climate chambers under the same conditions.

### Treatment of plants with feeding larvae

Larval hatching from eggs is close when the sclerotised, black head capsule of a larva inside the egg is visible from outside as a black spot. Hence, we observed eggs with such a black spot until larval hatching. As soon as the head of a neonate larva emerged from an egg laid on an *A. thaliana* leaf, the larva was immediately taken by a pair of soft tweezers before it started to feed on the egg shell or leaf tissue. Two batches of 20 larvae each were transferred to two leaves of a plant (i.e. 20 larvae per leaf, 40 larvae per plant) that had never had eggs (‘F’ treatment). Further two batches of 20 larvae each were transferred to two leaves of a plant on which these larvae had hatched from eggs (‘E+F’ treatment).

This procedure ensured that all tested larvae both on previously egg-laden plants and egg-free plants experienced the same experimental transfer by a pair of soft tweezers and that the same numbers of larvae were feeding on previously egg-laden and egg-free plants.

We used two different experimental set-ups to test whether the consumption of the egg shells by newly hatched larvae had an impact on the performance of the very young larvae (2-day-old) and their feeding activity.

- In the first set-up, larvae that had been removed from their hatching sites as described above, were replaced to this site and allowed to consume the egg shells by caging each batch of larvae at the oviposition site in a transparent clip-cage (2 cm diameter ×1.7 cm high) with bottom and top covered with mesh.
- In the second set-up, the larvae that had been removed from their hatching sites as described above, were replaced only next to this site, and were prevented to gain access to the egg shells until we recorded their weight 2 days after hatching. We caged the newly emerged larvae in the same type of clip-cages as described above, but placed the clip-cage close to the oviposition site without contact to the egg shells.

### Insect performance experiments

In order to elucidate the effects of prior egg deposition on *A. thaliana* on the performance of *P. brassicae* larvae, we used the same experimental setup as described above for the feeding treatments and compared performance of larvae that initiated feeding on egg-laden leaves with performance of larvae which started their larval feeding activity on an egg-free plant. We recorded the survival rate (P1) and weight of larvae (P2) after 2 days of feeding on egg-free or previously egg-laden plants. Additionally, we measured the leaf area that had been consumed by the young larvae after 2 days of feeding upon egg-free or previously egg-laden leaves.

After 2 days of feeding, the clip-cages were removed from the leaves, and the larvae were allowed to move and feed freely on the entire plant for a further 2 days. After these 4 days of larval feeding, plants were heavily damaged. In nature, larvae would start to leave the plant and search for another one.

To test whether feeding for 4 days on previously egg-laden plants affects later performance of larvae which moved to another plant, batches of ten 4-day-old larvae were transferred from both the egg-free plants and the previously egg-laden ones to undamaged, egg-free control plants where they remained until pupation. From these larvae the survival rates from the 4^th^ day after larval hatching until pupation were recorded. Performance parameters P1 and P2 were recorded from larvae on 8 plants ( = 8 replicates with 40 larvae per plant for each treatment: ‘F’, ‘E+F’) and parameter P3 from larvae on four plants initially ( = 4 replicates with 10 larvae per plant for each treatment: ‘F’, ‘E+F’).

### Sampling of leaf tissue

Leaf tissue samples (3 cm^2^) for analysis of glucosinolate concentrations and gene transcript levels were harvested from *A. thaliana* leaves of each treatment type (‘C’, ‘E’, ‘F’, or ‘E+F’). To study the effect of egg deposition just before larval hatching (treatment ‘E’), we collected leaves from egg-laden plants 5 days after egg deposition and excised pieces right next to the oviposition sites. Leaf pieces of the same size were taken from untreated (egg-free) control plants (‘C’). In order to obtain leaf samples from feeding-induced plants (‘F’ or ‘E+F’), samples were taken right next to the clip cage 2 days after larval feeding had started. Feeding-damaged leaf tissues were taken from the experiments where larvae had no contact to the egg shells. From each plant one leaf tissue sample was collected for the glucosinolate analysis and one for quantitative real-time PCR analysis. All samples were immediately transferred to liquid nitrogen and stored at −75°C until use.

### Glucosinolate (GLS) analysis

The GLS analysis followed the protocol described by Burow et al. [36]. http://www.plantcell.org/cgi/content/full/21/3/910?ijkey=2zrzQMXUWLqGzWx&keytype=ref – BIB11Lyophilised leaf tissue (5 mg) was ground, and GLS were extracted with 1.5 ml 80% methanol (v:v) containing the internal standard 4-hydroxybenzyl GLS for 5 min. After centrifugation (2500×g for 10 min), the supernatants were loaded onto a DEAE-Sephadex column. The column was washed with 1 ml 80% methanol (v:v) and 1 ml water. GLS were desulfated with 50 µl sulfatase solution overnight. After elution from the column, desulfoglucosinolate extracts were separated by HPLC on an Agilent HP1100 Series instrument equipped with a reverse-phase C-18 column (LiChrospher RP18ec, 250×4.6 mm, 5 μm particle size) and quantified by UV absorption at 229 nm relative to the internal standard using previously computed response factors [36]. Identity of intact GLS and of desulfoglucosinolates in the plant extracts was confirmed by liquid chromatography–mass spectrometry on a Bruker Esquire 6000 ion trap mass spectrometer (Bruker Daltonics).

### RNA extraction, cDNA synthesis and quantitative real-time PCR

Individual leaf samples were ground in liquid nitrogen, and total RNA was isolated using a hot-phenol extraction method [37]. Subsequently residual genomic DNA was removed by DNAse I treatment (Fermentas, St. Leon-Rot, Germany). Absence of genomic DNA was verified by qRT-PCR on total RNA with intron-specific primers of gene *At5g65080*. cDNA was synthesised from 2 μg of total RNA using Superscript™ III reverse transcriptase (Invitrogen). The efficiency of cDNA synthesis was estimated by qRT-PCR using primer pairs at the 3′- and 5′- ends of gene *At1g13440*. cDNA synthesis was rated satisfactory at (C_T__[5′ region]_ – C_T__[3′region]_) values ≤2.5. Concentrations of cDNAs were normalised to the transcript abundance of a housekeeping gene transcript (*UBQ10*; *At4g05320*).

qRT-PCR was conducted on an ABI 7500 Fast Real-Time PCR System (Applied Biosystems) similarly as described by Caldana et al. [38]. Briefly, amplifications were performed in a volume of 10 μl containing 1.0 μl of cDNA, 500 nM of each gene specific primer and 5.0 µl of SYBR Green master mix (Applied Biosystems). qRT-PCR cycles followed the thermal profile: 10 min 95°C – (15 sec 95°C – 60 sec 60°C) ×40. Specificity and quality of amplifications were tested by melting curve analyses and 4% agarose gel electrophoresis. Primary data analysis was done with SDS 2.2.1 software (Applied Biosystems). In each PCR run the *UBQ10* transcripts were measured in two technical replicates as calibrator samples. Target gene expression data were normalised by calculating the ΔC_T_  =  C_T (target gene)_ – C_T (UBQ10)_ value. To quantify the relative changes in gene expression in a treatment group relative to a reference group (untreated control or another treatment), we used the 2^−ΔΔCT^ method, where ΔΔC_T_  =  ΔC_T (treatment)_ – ΔC_T (reference)_ [39]. All primer sequences are listed in Table S2.

### Statistics

To prevent pseudo-replication, we averaged each larval performance parameter per plant prior to analysis. Furthermore, ‘E+F’ and ‘F’ treated plants within each replicate had larvae from the same parents. To control for parental effects on larval performance, we analysed the larval performance parameters using a repeated measures analysis of variance (rANOVA) with prior egg deposition as a within-subject factor and access to egg shells as a between-group factor. The number of replicates (*N*) per performance parameter is given in Table 1.

Differences in GLS concentrations were evaluated by means of a multivariate analysis of variance (MANOVA) followed by Fisher's LSD test for *post hoc* comparisons. GLS concentrations were log-transformed before the analysis. qRT-PCR data were analysed by analyses of variance (ANOVA) followed by Fisher's LSD test for *post hoc* comparisons. The exact number of replicates for the comparisons is given in the respective tables and figures (for GLS results: Table 3; for qRT-PCR results: Figure 1, Table S1). All statistical analyses were conducted using Statistica 10 (StatSoft, Inc.).

## Acknowledgments

We thank Michael Reichelt, Max Planck Institute for Chemical Ecology, for assistance in glucosinolate analysis, and Ute Braun, Freie Universität Berlin, for help in growing the plants and rearing the insects.

## Supporting Information

Table S1 **Transcript levels of genes involved in the regulation, biosynthesis and activation of glucosinolates in** ***Arabidopsis thaliana*** **Col-0 plants after different oviposition and feeding treatments.**
(DOCX)

Table S2 **Primers used for gene expression analysis.**
(DOCX)
