# Supplementary material for: Egg Laying of Cabbage White Butterfly (Pieris brassicae) on Arabidopsis thaliana Affects Subsequent Performance of the Larvae
Source: PLoS One. 2013 Mar 19;8(3):e59661. doi: 10.1371/journal.pone.0059661 (PMC3602411; doi:10.1371/journal.pone.0059661)
Supplement: Table S1 — Transcript levels of genes involved in the regulation, biosynthesis and activation of glucosinolates in Arabidopsis thaliana Col-0 plants after different oviposition and feeding treatments. (DOCX) [file pone.0059661.s001.docx]

**Table S1. Transcript levels of genes involved in the regulation, biosynthesis and activation of glucosinolates in *Arabidopsis thaliana* Col-0 plants after different oviposition and feeding treatments.**

|  | Gene | AGI Code | Description | Gene Expression^a^ | | | | |
| --- | --- | --- | --- | --- | --- | --- | --- | --- |
|  |  |  |  | E^b^ | F^b^ | E+F^b^ | [E+F]/F | [E+F]/E |
| *Regulation of Glucosinolate Biosynthesis* | | | |  |  |  |  |  |
|  | HAG1/MYB28 | At5g61420 | R2R3-MYB transcription factor 28 | 1.0 | 0.9 | 0.6 | 0.7 | 0.6 |
|  | HAG3/MYB29 | At5g07690 | R2R3-MYB transcription factor 29 | 1.2 | 1.2 | 1.0 | 0.8 | 0.8 |
|  | ATR1/MYB34 | At5g60890 | R2R3-MYB transcription factor 34 | 1.3 | 1.3 | 1.6 | 1.2 | 1.2 |
|  | HIG1/MYB51 | At1g18570 | R2R3-MYB transcription factor 51 | 1.0 | 0.7 | 0.5 | 0.7 | 0.5 |
|  | HIG2/MYB122 | At1g74080 | R2R3-MYB transcription factor 122 | 0.4 | 0.6 | 0.8 | 1.4 | 2.1 |
| *Glucosinolate Biosynthesis* | | |  |  |  |  |  |  |
|  | Aconitase | At2g43100 | Aconitase | 1.3 | 1.1 | 0.9 | 0.8 | 0.7 |
|  | BCAT4 | At3g19710 | Branched-chain aminotransferase 4 | 1.1 | 1.4 | 1.3 | 0.9 | 1.1 |
|  | MAM1 | At5g23010 | Methylthioalkylmalate synthase 1 | 1.2 | 1.3 | 1.1 | 0.8 | 0.8 |
|  | MAM3 | At5g23020 | Methylthioalkylmalate synthase 3 | 0.6 | 1.2 | 1.4 | 1.1 | 2.4 |
|  | CYP79F1 | At1g16410 | Cytochrome P450 79F1 | 1.2 | 1.2 | 0.9 | 0.8 | 0.8 |
|  | CYP79F2 | At1g16400 | Cytochrome P450 79F2 | 1.1 | 1.3 | 1.1 | 0.8 | 1.0 |
|  | CYP79B2 | At4g39950 | Cytochrome P450 79B2 | 0.9 | 1.1 | 0.9 | 0.8 | 1.0 |
|  | CYP79B3 | At2g22330 | Cytochrome P450 79B3 | 1.1 | 1.3 | 1.2 | 0.9 | 1.0 |
|  | CYP83A1 | At4g13770 | Cytochrome P450 83A1 | 1.0 | 1.1 | 1.1 | 1.0 | 1.1 |
|  | CYP83B1 | At4g31500 | Cytochrome P450 83B1 | 1.1 | 0.9 | 0.9 | 1.0 | 0.8 |
|  | SUR1 | At2g20610 | SUPERROOT 1 / C-S lyase | 1.1 | 0.9 | 0.8 | 0.9 | 0.7 |
|  | UGT74B1 | At1g24100 | UDP-glucose thiohydroximate S-glucosyltransferase | 1.4 | 1.1 | 1.0 | 0.9 | 0.7 |
|  | GSH1 | At4g23100 | Glutamate-cysteine ligase | 1.3 | 1.1 | 1.0 | 0.9 | 0.7 |
|  | FMO_GS-OX2_ | At1g62540 | Flavin-monooxygenase glucosinolate S-oxygenase 2 | 1.3 | 2.3** | 1.0 | 0.4** | 0.8 |
|  | FMO_GS-OX5_ | At1g12140 | Flavin-monooxygenase glucosinolate S-oxygenase 5 | 1.1 | 0.7* | 0.6** | 0.9 | 0.5** |
|  | CYP81F2 | At5g57220 | Cytochrome P450 81F2 | 1.4 | 0.7 | 0.8 | 1.1 | 0.6 |
| *Glucosinolate Activation* | | |  |  |  |  |  |  |
|  | ESM1 | At3g14210 | Epithiospecifier modifier 1 | 1.2 | 0.7 | 0.6 | 0.8 | 0.5 |
|  | ESP | At1g54040 | Epithiospecifier protein | 1.3 | 1.6 | 1.1 | 0.6 | 0.8 |
|  | NSP1 | At3g16400 | Nitrile-specifier protein 1 | 1.0 | 5.0*** | 3.7** | 0.7 | 3.5** |
|  | NSP3 | At3g16390 | Nitrile-specifier protein 3 | 0.9 | 2.9*** | 3.2** | 1.1 | 3.4** |
|  | NSP5 | At5g48180 | Nitrile-specifier protein 5 | 1.2 | 1.7 | 1.3 | 0.8 | 1.1 |
|  | PEN2 | At2g44490 | PENETRATION 2 / Myrosinase | 1.4 | 1.2 | 0.9 | 0.7 | 0.7 |
|  | PEN3 | At1g59870 | PENETRATION 3 / Myrosinase | 1.4 | 1.2 | 1.0 | 0.8 | 0.7 |
|  | TGG1 | At5g26000 | Thioglucoside glucohydrolase 1 / Myrosinase | 1.0 | 0.8 | 0.5 | 0.5 | 0.5 |
|  | TGG2 | At5g25980 | Thioglucoside glucohydrolase 2 / Myrosinase | 2.0 | 0.9 | 0.8 | 0.9 | 0.4 |

E: leaves on which eggs were laid and left for 5 days; F: leaves that never had eggs but were fed on by *Pieris brassicae* caterpillars for 2 days; E+F: leaves on which eggs were laid and caterpillars hatched from eggs and fed for 2 days.

^a^ Gene expression = 2^-(Δct sample – Δct reference)^

^b^ relative to untreated controls

* *P* < 0.05; ** *P* < 0.01; *** *P* < 0.001; ANOVA with Fisher’s LSD test for *post-hoc* comparisons; N = 4-8.
