# Supplementary material for: Egg Laying of Cabbage White Butterfly (Pieris brassicae) on Arabidopsis thaliana Affects Subsequent Performance of the Larvae
Source: PLoS One. 2013 Mar 19;8(3):e59661. doi: 10.1371/journal.pone.0059661 (PMC3602411; doi:10.1371/journal.pone.0059661)
Supplement: Table S2 — Primers used for gene expression analysis. (DOCX) [file pone.0059661.s002.docx]

**Table S2. Primers used for gene expression analysis.**

| **Gene** | **AGI Code** | **Forward Primer** | **Reverse Primer** |
| --- | --- | --- | --- |
| MAF5 | At5g65080 | TTTTTTGCCCCCTTCGAATC | ATCTTCCGCCACCACATTGTAC |
| GAPC2 (3'-end) | At1g13440 | TCTCGATCTCAATTTCGCAAAA | CGAAACCGTTGATTCCGATTC |
| GAPC2 (5'-end) | At1g13440 | TTGGTGACAACAGGTCAAGCA | AAACTTGTCGCTCAATGCAATC |
| UBQ10 | At4g05320 | GGCCTTGTATAATCCCTGATGAATAAG | AAAGAGATAACAGGAACGGAAACATAGT |
| Aconitase | At2g43100 | AACATGCTCCGGTCTGTCTTGG | TGCGTAAGACTCAGCAACTATGGC |
| ATR1/MYB34 | At5g60890 | AGACCCGACATTAAGAGAGGAGAG | ATTGCGGCCCACTTGTTACC |
| BCAT4 | At3g19710 | TCGTGATTTCGGCTACCAGGTC | AGCTTCGTCCGCATCGAGAAAG |
| CYP79B2 | At4g39950 | TCAAACCCACCATTAAGGAGCTTG | TTCCACGGCGTTTGATGGATTG |
| CYP79B3 | At2g22330 | ACCGGAAAGAGAGGATGTGCTG | CGCTAGCATCATGGTCGTTATCGC |
| CYP79F1 | At1g16410 | CATTATGTCCCTTCCCATCTTGCG | ACATGAATGTGGCTACCTTTGGG |
| CYP79F2 | At1g16400 | CCTCATGTTGCCCGTCAAGATACC | ACGCTCCGGTTCGTATGCTAATG |
| CYP81F2 | At5g57220 | CATCATCAAAGGGCTCATGCTCAG | AATGTTACGGCCGCAGTATCCG |
| CYP83A1 | At4g13770 | AAGAGAGTCAAGCCCGAAACCG | GTTCCCGCCACTACAATATCCAAG |
| CYP83B1 | At4g31500 | TGGATATTGTTGTGCCGGGAACTG | ACACTCCTCACTTCGTCTTGAGC |
| ESM1 | At3g14210 | GCGCCGGTTGAATCTATGACAC | TTTCTCGATGTAGCTGTCGTTCC |
| ESP | At1g54040 | TGAATACTCCCACGCGGTTCAG | AGCTGAGCCCATTTCCCATCAG |
| FMO_GS-OX2_ | At1g62540 | GCCGTTGTTGTTTGCAGTGGAC | ATTTCCGATGACCACCACCACCTC |
| FMO_GS-OX5_ | At1g12140 | CTTCATCGGTTTACCCTGGATGAC | GCCTCACGCTTTGCATAGTAGG |
| GSH1 | At4g23100 | TGTCCTGAAACTCGCAAAGGATG | CATCGACTGCGTTCAAGAAACCG |
| HAG1/MYB28 | At5g61420 | AGACTGCGATGGACCAACTACC | TCTCGCTATGACCGACCACTTG |
| HAG3/MYB29 | At5g07690 | AGTTGTAGATTGCGATGGGCTAAC | TGTCTCGCTATGACTGACCACTTG |
| HIG1/MYB51 | At1g18570 | CCTTCACGGCAACAAATGGTCTG | TACCGGAGGTTATGCCCTTGTG |
| HIG2/MYB122 | At1g74080 | GAGAGTTTAGCCAAGACGAGGAAG | CTATGGCCGACCATTTGTTGCC |
| MAM1 | At5g23010 | AAGTGGCAATGCGTCGCTTGAG | AGCCCGTGTACTCTTGAACCATC |
| MAM3 | At5g23020 | TGCTACCGCCAACACAATATCCG | ACATTTCAAAGCCATCACGACCTC |
| NSP1 | At3g16400 | TGCTTGGGAAGTGGATTAAGGTG | GGGCTGATTTGGTGTGAACTCG |
| NSP3 | At3g16390 | GCGCGATTTGATCATGACCGTTTG | CATCACACAGACACACAGACACAC |
| NSP5 | At5g48180 | TGTTTCCGGCGGTTTCTTACGG | TAAGCTCATGCGGCTCTTCCTC |
| PEN2 | At2g44490 | TGCGCATAATCCACTTTGGTACG | AGTCGGATGCTGATGCCAAC |
| PEN3 | At1g59870 | ACTGCAGATGGGAACAGAAGAGG | TTCTTATTGCCTGCACCACCTG |
| SUR1 | At2g20610 | CCGGCAAAGGCAATTCTTACGG | TCATATAATCAGCAACGGCTCGTC |
| TGG1 | At5g26000 | ATGGACTCACGCACAACTCTCAC | AGTAACTGGCTGCATTGAACGG |
| TGG2 | At5g25980 | CCGCAAGGCCATCAAGTGAAAG | AATCTGACGGTGTAGCCGTTGC |
| UGT74B1 | At1g24100 | ACGTTGGAAGGGTTGAGTTTGGG | TCTGATCACTCCACTGAGGCACAC |
